# Observation of Histone H2AX Phosphorylation by Radiation-Induced Bystander Response Using Titanium Characteristic X-ray Microbeam

**DOI:** 10.3390/biology12050734

**Published:** 2023-05-18

**Authors:** Masanori Tomita, Masaya Torigata, Tadayuki Ohchi, Atsushi Ito

**Affiliations:** 1 Biology and Environmental Chemistry Division, Sustainable System Research Laboratory, Central Research Institute of Electric Power Industry (CRIEPI), Komae, Tokyo 201-8511, Japan; 2 School of Engineering, Tokai University, Hiratsuka, Kanagawa 259-1292, Japan; torigata.masaya@qst.go.jp (M.T.); aeito@keyaki.cc.u-tokai.ac.jp (A.I.); 3 NTT Advanced Technology Co., Atsugi, Kanagawa 243-0124, Japan; tadayuki.ohchi@ntt-at.co.jp

**Keywords:** microbeam, X-rays, radiation-induced bystander response, DNA double-strand break, DNA repair

## Abstract

**Simple Summary:**

X-ray microbeams are useful tools for elucidating the mechanisms underlying non-target effects, such as the radiation-induced bystander response (RIBR) that occurs under heterogeneous exposure conditions. The microbeam X-ray cell irradiation system at the Central Research Institute of Electric Power Industry (Tokyo, Japan) has been upgraded to enable irradiation with titanium characteristic X-rays (Ti_K_ X-rays), which have a longer penetration distance than aluminum characteristic X-rays. The beam size of the Ti_K_ X-rays was elliptical, with a long diameter of 7.5 μm and a short diameter of 6.9 μm. The dose rate at the sample position was approximately 0.8 Gy/min. Using this system, we irradiated the nuclei of HeLa cells with high precision and then analyzed RIBR. The percentage of bystander cells with pan-nuclear induction of phosphorylated histone H2AX on serine 139 (γ-H2AX) was significantly increased in one field of view, including microbeam-irradiated cells 180 and 360 min after Ti_K_ X-ray microbeam irradiation.

**Abstract:**

Radiation-induced bystander response (RIBR) is a response induced in non-irradiated cells that receive bystander signals from directly irradiated cells. X-ray microbeams are useful tools for elucidating the mechanisms underlying RIBR. However, previous X-ray microbeams used low-energy soft X-rays with higher biological effects, such as aluminum characteristic X-rays, and the difference from conventional X-rays and γ-rays has often been discussed. The microbeam X-ray cell irradiation system at the Central Research Institute of Electric Power Industry has been upgraded to generate higher energy titanium characteristic X-rays (Ti_K_ X-rays), which have a longer penetration distance sufficient to irradiate 3D cultured tissues. Using this system, we irradiated the nuclei of HeLa cells with high precision and found that the pan-nuclear induction of phosphorylated histone H2AX on serine 139 (γ-H2AX) in the non-irradiated cells increased 180 and 360 min after irradiation. We established a new method to quantitatively evaluate bystander cells, using the fluorescence intensity of γ-H2AX as an indicator. The percentage of bystander cells increased significantly to 23.2% ± 3.2% and 29.3% ± 3.5% at 180 and 360 min after irradiation, respectively. Our irradiation system and the obtained results may be useful for studies of cell competition as well as non-targeted effects.

## 1. Introduction

Comprehending the biological effects of low-energy transfer (LET), low-dose, and low-dose-rate ionizing radiation is essential for the optimization of the system of radiation protection. Non-targeted effects (or non-DNA-targeted effects) are defined as a paradigm in radiobiology and are an indirect effect of intra- and inter-cellular communications involving targeted and non-targeted cells [1,2]. Radiation-induced bystander response (RIBR) is generally defined as a response induced in non-irradiated cells that receive bystander signals from directly irradiated cells within an irradiated cell population [1], representing the non-target effect. RIBR came to attention in 1992 with the findings of Nagasawa and Little [3]. In their study, the frequency of sister chromatid exchange (SCE) in Chinese hamster ovary cells was significantly increased at doses as low as 0.31 mGy. At this dose, 30% of the cells showed an increase in SCE frequency, but less than 1% of the nuclei were passed by α particles. The heterogeneity of radiation doses at the cellular level is a major barrier to understanding the biological mechanisms under low-dose and low-dose-rate irradiation conditions. Therefore, the development of microbeam irradiation systems that can precisely target individual cells or tissues and generate non-uniform irradiation conditions that simulate those under low-dose and low-dose-rate irradiation has been promoted worldwide [1,2,4,5]. Several high-LET charged-particle microbeam irradiation facilities have been developed worldwide and have revealed important mechanisms related to radiation-induced bystander responses [2,4,5]. For example, NF-κB-, cyclooxygenase-2 (COX-2)-, and Akt-mediated signaling pathways are involved in α-particle-induced bystander response [6]. We also showed that DNA double-strand breaks (DSBs) and reproductive cell death are induced by nitric oxide (NO) in non-irradiated normal human fibroblasts due to high-LET heavy-ion-induced bystander signaling. NF-κB, Akt, and COX-2 are key molecules in the NO-mediated bystander signaling pathway, methodically activated depending on an incubation time after irradiation [7].

However, few X-ray microbeam irradiation facilities are still available for biological experiments. The Gray Cancer Institute (Northwood, UK) is a pioneer in this research field and was the first in the world to successfully develop a system that can irradiate cells with a focused carbon K-shell (278 eV) ultrasoft X-ray microbeam [8,9]. In Japan, the synchrotron radiation (SR) X-ray microbeam irradiation system has been developed at the Photon Factory, High Energy Accelerator Research Organization (PF, KEK, Ibaraki, Japan) [4,10,11] and is still used in various biological studies. The microbeam X-ray cell irradiation system at the Central Research Institute of Electric Power Industry (CRIEPI, Tokyo, Japan) [4,12] has also been successfully developed as a tabletop type system. In this system, aluminum characteristic X-rays (Al_K_ X-rays, 1.49 keV) are generated by the focused electron bombardment of an aluminum target and are focused through the Fresnel zone plate (FZP). The minimal beam size of Al_K_ X-rays, which was measured through knife-edge scanning, was 1.8 μm in diameter [4,12].

Using these X-ray microbeam irradiation systems, the dose-response of the bystander cell-killing effect of confluent normal diploid human lung fibroblast WI-38 cells has been investigated [1,12,13]. It shows a biphasic relationship with irradiation dose when the nuclei of five cells at the center of a dish are irradiated. The surviving fraction (SF) significantly decreases at irradiation doses of >0.09 Gy and is 0.85 at 1.4 Gy after exposure to a 5 μm × 5 μm SR X-ray microbeam [1,13]. However, the SF reaches approximately 1.0 at 1.9 and 4.7 Gy. At 9.3 Gy, cell survival decreases again. Al_K_ X-ray microbeams at doses of 0.12 and 0.23 Gy do not provide significant bystander cell-killing effects. The SF decreases significantly at doses of ≥0.47 Gy and is 0.88 at 1.2 Gy, but the decrease is partially suppressed between doses 2.3 and 7.0 Gy. At doses of >14 Gy, cell survival decreases and reaches a plateau. Furthermore, in mutated p53 cells, the SF decreases sharply up to 1 Gy and remains low up to 5 Gy [14]. Bystander cell killing effects are mediated by NO. Thus, the suppression of the bystander cell-killing effect at doses of approximately 2–7 Gy is mainly caused by the activated function of wild-type p53 and Nitric Oxide Synthase (NOS).

“Cell competition” has attracted attention as a biological effect that occurs under heterogeneous exposure conditions. Cell competition, based on the comparison of relative cell fitness between neighboring cells, is a prominent example of tissue adaptability [15]. It has been suggested that cell competition could play a central role in error correction during development and in cancer progression. An X-ray microbeam with sufficient penetrating length to irradiate cells is necessary to analyze cell competition in 3D cultured tissues, such as organoids and spheroids. The attenuation length (1/e) of Al_K_ X-rays in water is approximately 7.1 μm and can penetrate only one cell layer [16]. On the other hand, the 1/e attenuation length of titanium characteristic X-rays (Ti_K_ X-rays, 4.51 keV) in water is approximately 171 μm and can penetrate a certain thickness of 3D cultured tissues.

Previous X-ray microbeams used low-energy soft X-rays with higher biological effects, such as aluminum characteristic X-rays, and the difference between conventional X-rays and γ-rays has been widely discussed. We upgraded our microbeam X-ray irradiation system [4,12] to enable irradiation with higher energy Ti_K_ X-ray microbeams in addition to Al_K_ X-ray microbeams. We evaluated the performance of the Ti_K_ X-ray microbeam and showed that it is sufficient for the targeted irradiation of cells. In addition, we found that the pan-nuclear induction of phosphorylated histone H2AX on serine 139 (γ-H2AX)—a surrogate marker for DSBs—in the non-irradiated cells increased after irradiation. Therefore, we established a new method to quantitatively evaluate bystander cells using the fluorescence intensity of γ-H2AX as an indicator. The percentage of bystander cells increased significantly at 180 and 360 min after irradiation. Our irradiation system and the obtained results may be useful for studies of cell competition as well as non-targeted effects.

## 2. Materials and Methods

### 2.1. Cell Culture

Normal diploid human lung fibroblast WI-38 cells were obtained from the American Type Culture Collection (ATCC, Manassas, VA, USA). Human cervical cancer HeLa cells were obtained from RIKEN BioResource Center (Ibaraki, Japan). The cells were cultured in Dulbecco’s Modified Eagle’s medium (DMEM) and Ham’s F-12 Nutrient Mixture (DMEM/F-12) medium (Sigma-Aldrich, St. Louis, MO, USA) supplemented with 10% fetal bovine serum (Hyclone Laboratories, Inc., Logan, UT, USA), penicillin, and streptomycin (Gibco, Grand Island, NY, USA). The cells were maintained at 37 °C in a humidified incubator in a 95% air/5% CO_2_ atmosphere.

### 2.2. Microbeam Irradiation

#### 2.2.1. Microbeam X-ray Cell Irradiation System

**Figure 1:**
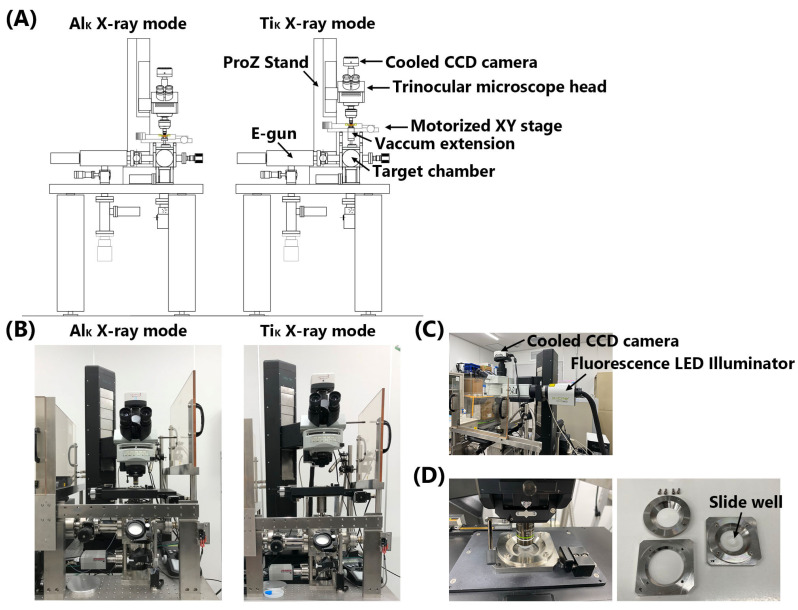
Upgraded microbeam X-ray cell irradiation system. (**A**) Schematic of the Al_K_ and Ti_K_ X-ray modes of the irradiation system. (**B**) Al_K_ and Ti_K_ X-ray modes of the irradiation system. (**C**) Cooled CCD camera and fluorescence LED illuminator set on the trinocular microscope head. (**D**) Cell irradiation dish set on the motorized XT stage (**left**) and its components (**right**). Cells were observed using a water-dipping lens. The slide well was cut to form a doughnut shape and attached to a polypropylene film to limit the cell adhesion surface.

The microbeam X-ray cell irradiation system at CRIEPI (Tokyo, Japan) was used in this study (Figure 1) [4,12]. The system was upgraded to enable irradiation with Ti_K_ X-rays in addition to Al_K_ X-rays (Figure 1A–C). A lanthanum hexaboride (LaB_6_) filament electron gun (E-gun, OME-3040LA, Omegatron, Ibaraki, Japan) was operated at voltages up to −30 kV relative to the target. The electron beam was focused onto the surface of the Ti or Al target (length 20 mm × width 10 mm × height 1 mm) by using an electromagnetic lens. Bremsstrahlung X-rays with higher energy, which are also generated with characteristic radiation, were removed by reflection with a grazing incidence mirror, as described previously [9]. A new FZP specially designed for focusing Ti_K_ X-rays was manufactured by the NTT Advanced Technology Corporation (Tokyo, Japan). The FZP was 123.6 μm in diameter and 18 μm in focal length. The center stop diameter and outermost zone width of the FZP were 4.5 μm and 36 nm, respectively. The distance from the light source to the FZP was 198 mm for the Ti_K_ X-rays and approximately 150 mm for the Al_K_ X-rays. A vacuum extension was inserted into the Ti_K_ X-ray mode to fill this gap (Figure 1A,B). However, the motorized XY stage and trinocular microscope head (Olympus, Tokyo, Japan) were positioned higher than those in the Al_K_ X-ray mode to allow the insertion of the vacuum extension. A Pro Z stand (Prior Scientific, Cambridge, UK) was used to move the trinocular microscope head upward and downward. An order-selecting aperture (OSA) was used to select first-order diffracted Ti_K_ or Al_K_ X-rays by blocking unwanted zero and higher-order X-rays. The OSA consisted of a 30 μm diameter pinhole [4,12]. A high-precision positioning motorized XY stage (H117E1B4, Prior Scientific) and Pro Z stand were controlled using a ProScan III controller (Prior Scientific). A high-resolution, 1.4 mega-pixel, monochrome, cooled CCD camera (INFINITY3-1M, Teledyne Lumenera, Ontario, Canada) and an X-Cite 120LED Boost High-Power LED illumination System (Excelitas Technologies, Waltham, MA, USA) were combined with a trinocular microscope head (Figure 1C). Image-Pro 10 software (Media Cybernetics, Rockville, MD, USA) was used for image analysis. The irradiation software running on Image Pro 10 was the same as that used in the SR X-ray microbeam irradiation system at PF, KEK [4,10,11], but was modified to run on Windows 10.

#### 2.2.2. Microbeam Irradiation

**Figure 2:**
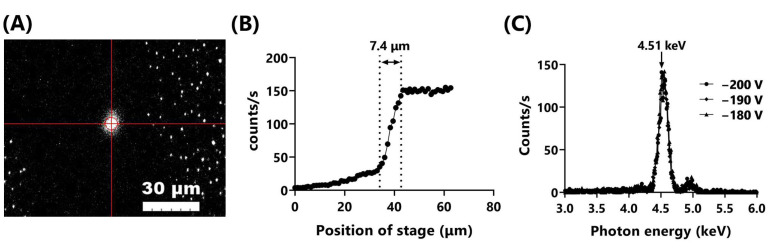
Ti_K_ X-ray microbeams generated by the Ti_K_ X-ray mode of the microbeam X-ray cell irradiation system. (**A**) Ti_K_ X-ray microbeams visualized with the scintillator. Bar represents 30 μm. (**B**) Output of Ti_K_ X-rays was measured by knife-edge scanning using the X-ray detector set on the motorized XY stage. Beam size was estimated to be 7.4 μm in diameter. (**C**) Measurements of the energy spectrum were acquired at the sample position through the OSA by adjusting the bias voltage (VB; −180, −190, and −200 V).

To irradiate cells with X-ray microbeams, custom-designed dishes (34 mm in diameter) [4,10,11] were prepared as shown in Figure 1D, with the bottom culture surface composed of a 3 μm-thick polypropylene film (Toray Industries, Inc., Tokyo, Japan). The cell adhesion surface was limited by cutting the slide well (1 cm diameter, 0.50 mm thick adhesive well sticker, Diversified Biotech, Dedham, MA, USA) to form a doughnut shape and attaching it to the polypropylene film. The film surfaces in the wells were coated with fibronectin (Sigma-Aldrich). The cells were cultured in these dishes for 24 h before irradiation. Cell nuclei were stained with a 1 μM Hoechst 33258 solution (DOJINDO Laboratories, Kumamoto, Japan) for 30 min. After two washes with phosphate-buffered saline (PBS), the cells were incubated in 5 mL of fresh medium for irradiation. To irradiate the cells, the beam position was detected using a scintillator (CaF2(Eu), OHYO KOKEN KOGYO, Tokyo, Japan) that recorded the coordinates of the centers of the X-ray microbeams (Figure 2A). The positions of the cell nuclei were determined based on the fluorescent images of the cell nuclei obtained using a CCD camera. The positions of the targets and the exposure period were controlled using the irradiation software. The number of photons was measured using an X-ray detector, XR-100CR (Amptek, Bedford, MA, USA). The absorbed dose was calculated as previously reported [12,13].

### 2.3. Immunofluorescence Microscopy

After irradiation, the HeLa cells were washed three times with PBS on ice, fixed with 4% paraformaldehyde in PBS for 20 min, and then permeabilized for 10 min with 0.1% Triton X-100 in PBS at room temperature. After rinsing with PBS supplemented with 0.01% Tween-20 (T-PBS), the cells were blocked with 10% bovine serum albumin (BSA) (NACALAI TESQUE, Inc., Kyoto, Japan) in PBS at room temperature for 20 min or 4 °C for several hours. After washing with T-PBS once, the cells were incubated overnight at 4 °C with 1% BSA in T-PBS containing anti-γ-H2AX (05-636, Merck, Darmstadt, Germany) antibodies diluted at 1:500. The dishes were rinsed three times with T-PBS and then incubated at room temperature for 1 h with PBS containing Alexa-594-conjugated anti-mouse IgG (A-11032, Invitrogen, Eugene, OR, USA) diluted at 1:400. The dishes were washed twice with T-PBS and once with PBS, and then mounted with SlowFade Diamond Antifade Mountant with DAPI (Invitrogen). WI-38 cells were fixed as described previously [13] and then incubated overnight at 4 °C with 1% BSA in T-PBS containing anti-γ-H2AX and anti-p53-binding protein 1 (53BP1) antibodies (PC712, Merck) diluted at 1:500. The dishes were rinsed three times with T-PBS and then incubated at room temperature for 1 h with PBS containing Alexa-488-conjugated anti-mouse IgG (A-11029, Invitrogen) and Alexa-594-conjugated anti-rabbit IgG (A-11012, Invitrogen) diluted at 1:400. The dishes were washed twice with T-PBS and once with PBS, and then mounted with SlowFade Diamond Antifade Mountant with DAPI. Immunofluorescence images of the cells were captured using a CCD camera on Image-Pro 10 and analyzed using ImageJ software (Rasband, W.S., ImageJ, U.S. National Institutes of Health, Bethesda, Maryland, MD, USA, https://imagej.nih.gov/ij/, 1997–2018 accessed on 2 November 2022).

### 2.4. Statistics

Statistical analyses were performed using Microsoft Excel and GraphPad Prism 9 software (GraphPad Software, Boston, MA, USA). Statistical significance was analyzed using Tukey’s multiple comparison tests and considered at *p* ≤ 0.05.

## 3. Results

### 3.1. Upgrade of the Microbeam X-ray Cell Irradiation System

The microbeam X-ray cell irradiation system [4,12] was upgraded to enable irradiation with Ti_K_ and Al_K_ X-rays (Figure 1A–C). The motorized XY stage, cooled CCD camera, irradiation program, and image analysis software were updated. Therefore, we irradiated the cells with Al_K_ X-ray microbeams in Al_K_ X-ray mode to determine whether the performance before the upgrade [4] was maintained. The nuclei of WI-38 cells were irradiated with 1–20 Gy, delivered using Al_K_ X-ray microbeams, and the cells were fixed 30 min after irradiation. The beam size under the present experimental conditions was determined to be approximately 4 μm in diameter based on the image of the beam visualized with a scintillator (Figure S1A). The dose rate was 1.4 Gy/s. As shown in Figure S1B, colocalization of 53BP1 and γ-H2AX, surrogate markers for DSBs, was observed within the targeted cell nuclei; however, foci formation of γ-H2AX was less obvious at doses ≤2 Gy. Maeda et al. [17] reported that the foci formation of γ-H2AX could not be detected following the SR X-ray microbeam irradiation of the nuclei of WI-38 cells and Chinese hamster V79 cells at doses ≤2 Gy, although 53BP1 accumulated at the target site in the nucleus even at a dose of 1 Gy. Conversely, γ-H2AX foci could be observed following whole-cell irradiation at doses ≥1 Gy. These results suggest that ataxia telangiectasia mutated (ATM)-mediated DNA damage responses (DDR) are modified with or without cytoplasmic irradiation at doses ≤2 Gy and nuclear-cytoplasmic shuttling of ATM [18] may be an important upstream moderator/mediator of the DDR.

Figure 2A shows an image of Ti_K_ X-ray microbeams visualized using a scintillator. The size and intensity of the microbeams were controlled by adjusting the VB. To minimize the beam size, we operated the system at a VB of −200 V and a filament current (IF) of 1600 mA at the indicated experimental conditions. The beam was elliptical, with a long diameter of 7.5 μm and a short diameter of 6.9 μm. The long diameter of the beam, measured more precisely by knife-edge scanning, was 7.4 μm (Figure 2B). This beam size was approximately four times larger than the 1.8 μm of Al_K_ X-ray microbeams [9] but small enough compared to the size of the mammalian cell nucleus (~10 μm in diameter). The energy spectrum was measured at the sample position through the OSA by adjusting the VB (−180, −190, and −200 V). The peaks of photon energy were approximately 4.51–4.52 keV, almost consistent with the 4.51 keV of the Ti-Kα X-ray (Figure 2C). The small peaks around 4.9 keV were Ti-Kβ X-rays. The dose rate at the sample position was approximately 0.8 Gy/min.

### 3.2. Induction of γ-H2AX-Positive Bystander Cells

**Figure 3:**
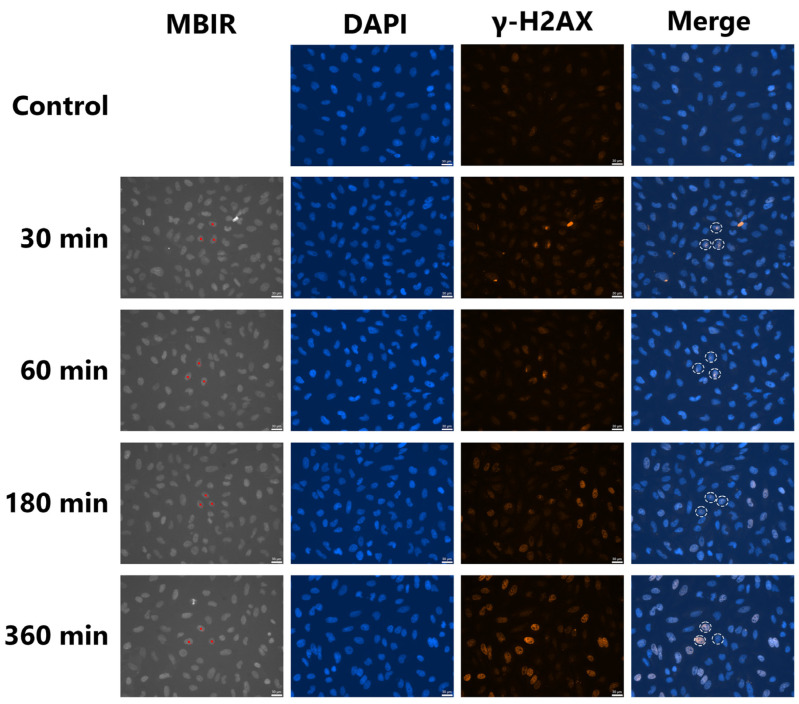
Accumulation of γ-H2AX in Ti_K_ X-ray microbeam-irradiated and non-irradiated HeLa cells. Three targeted cell nuclei were microbeam irradiated at 4 Gy. MBIR (microbeam irradiation) is a fluorescent image captured at the time of microbeam irradiation. Before irradiation, cell nuclei were stained with Hoechst 33258 solution for targeted irradiation of cell nuclei. The red squares are the irradiation positions set by the irradiation software. White dotted circles in the merged images indicate irradiated cell nuclei after fixation and immunofluorescent staining. Bars represent 30 μm.

**Figure 4:**
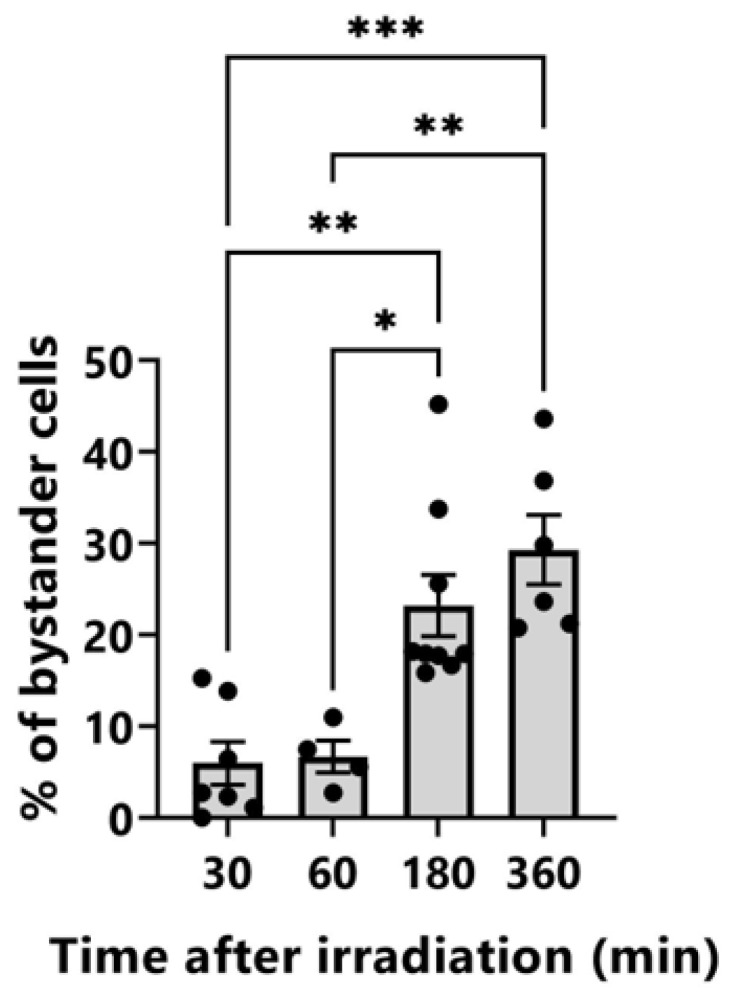
Induction of bystander cells after Ti_K_ X-ray microbeam irradiation of the nuclei of three cells in the center of the field of view at a dose of 4 Gy. Unirradiated HeLa cells with γ-H2AX fluorescence intensity higher than the average + σ of control cells were determined to be bystander cells. The error bars represent standard errors of the mean (SEM) based on the four to nine independent experiments. *p*-values (* *p* < 0.05, ** *p* < 0.01, *** *p* < 0.001) calculated with Tukey’s multiple comparison test.

HeLa cells were stained with Hoechst 33258 solution before irradiation, and fluorescent images of the cell nuclei were captured using a cooled CCD camera. The nuclei of three cells in the center of the field of view were irradiated with 4 Gy of Ti_K_ X-ray microbeams. No toxicity was detected as a result of the staining and short UV radiation exposures needed to obtain fluorescent images as described previously [13]. The cells were fixed at 30, 60, 180, and 360 min after irradiation. In the target irradiated cells, localized γ-H2AX induction was detected (Figure 3). The intensity of γ-H2AX tended to decrease 180 min after irradiation, although pan-nuclear induction of γ-H2AX was observed in two of the three cells 360 min after irradiation. Accumulation of γ-H2AX in irradiated cells disappeared more quickly after 2 Gy of irradiation. To distinguish between the cells after 360 min of irradiation, at least 4 Gy of irradiation was required. In the non-irradiated cells, the pan-nuclear induction of γ-H2AX increased at 180 and 360 min after irradiation. The distribution of fluorescence intensity of γ-H2AX in the non-irradiated control cells was analyzed using ImageJ to quantitively assess the induction of bystander γ-H2AX-positive cells (Figure S2). The fluorescence intensity was 35.01 ± 13.95. The cells with fluorescence intensities higher than 48.96 (average + σ) were determined to be bystander cells in the irradiated dishes. As shown in Figure 3, the nuclei of three cells in the center of the field of view were irradiated with 4 Gy of microbeams. The percentage of bystander cells in one field of view captured at the same X and Y coordinates as the irradiation is shown in Figure 4. The percentage of bystander cells increased significantly to 23.2% ± 3.2% and 29.3% ± 3.5% at 180 and 360 min after irradiation, respectively. These results indicate that bystander signals induced γ-H2AX accumulation in the non-irradiated cells in the vicinity of the Ti_K_ X-ray microbeam-irradiated cells.

## 4. Discussion

The microbeam X-ray cell irradiation system at CRIEPI was upgraded to enable irradiation with Ti_K_ X-rays, which have a longer penetration distance, in addition to Al_K_ X-rays. A comparison of Al_K_ X-ray and Ti_K_ X-ray microbeams is presented in Table 1. Ti_K_ X-ray microbeams have a wider beam width and a lower dose rate than Al_K_ X-ray microbeams. Therefore, multipoint and high-dose irradiation using Ti_K_ X-ray microbeams is difficult (Figure S1). However, the beam size is smaller than the nuclei of many mammalian cells, and the penetration distance is sufficiently long to irradiate cells in 3D cultured tissues. Several studies on RIBR in 3D tissue models irradiated with charged-particle microbeams have been reported [2,19,20,21]. Prise et al. [19] used a urothelial model based on a section of human or porcine ureter. The ureter is highly organized with four to five layers of urothelium. Sedelnikova et al. [20] used a respiratory tract model consisting of three to four layers of human-derived tracheal/bronchial epithelial cells and a full-thickness skin model. Based on our system, Fujimichi et al. [22] have recently used Ti_K_ X-ray microbeams for the targeted irradiation of single intestinal stem cells in an intestinal organoid. The irradiated cells divided and underwent cell death and fragmentation, and their debris was eliminated into the lumen. For the first time, we succeeded in capturing the moment when irradiated stem cells were eliminated within the organoids.

Pan-nuclear induction of γ-H2AX in the non-irradiated cells increased 180 and 360 min after irradiation (Figure 3 and Figure 4). This result was also observed in two of the three cells 360 min after irradiation. Foci formation of γ-H2AX is widely used as a surrogate marker for DSBs. Sokolov et al. [23] evaluated that the cells with four or more γ-H2AX foci per cell (≥4 fpc) were bystander cells. The bystander cells containing ≥4 fpc were 26% at 18 h after exposure of the targeted cells with 20 alpha particles. In the present study, to evaluate bystander cells inducing pan-nuclear γ-H2AX, we established a new method whereby the cells with fluorescence intensities higher than average + σ were determined to be bystander cells. The value of average + σ must be determined according to the experimental conditions (cell type, antibody, observation conditions, etc.). The percentage of bystander cells in this study was 23.2% ± 3.2% and 29.3% ± 3.5% at 180 and 360 min after irradiation, respectively. Although the time after irradiation at which bystander cells were detected differed, the percentage of the bystander cells was almost identical to that found by Sokolov et al. [23]. Our evaluation method is simpler and more quantitative than counting the number of γ-H2AX foci per cell and can be used for a more objective estimation of RIBR. However, further validation using cancer cell lines other than HeLa cells is needed in the future.

Pan-nuclear H2AX phosphorylation is also induced by UV-C irradiation (254 nm) [24]. It was observed in all phases of the cell cycle within 1 h and was the highest in the S phase. After UV irradiation, H2AX phosphorylation levels in G_1_ cells reached a maximum at 2 h and then decreased between 4 and 8 h. Therefore, the present results differ from those on H2AX phosphorylation induced by UV irradiation. Induction of pan-nuclear phosphorylation of H2AX has also been observed in cells irradiated with accelerated alpha particles [25] and heavy-ion irradiation [26]. The pan-nuclear response was not detected after X-ray irradiation and was driven primarily by ATM after charged particle irradiation. Recently, Moeglin et al. [27] reported that H2AX phosphorylation is systematically pan-nuclear in cancer cells, including HeLa cells, when stressed with replication stress-inducing drugs immediately before they die. However, these findings do not explain our results. Based on the results of X-ray microbeam irradiation [1,12,13,14,28,29], we previously suggested a possible model of photon-induced bystander signaling. We found that a chief initiator and/or mediator of photon-induced bystander responses is NO. Peroxynitrite, which is formed by the reaction between NO and reactive oxygen species, can induce DNA damage and increase the proportion of γ-H2AX-positive cells, even at 1 μM [30]. Although many studies have focused on the induction of γ-H2AX in bystander cells [1,2,23,31], further investigation of NO-mediated DNA damage induction is warranted to elucidate the mechanisms by which pan-nuclear γ-H2AX is induced in bystander cells.

In the present study, we analyzed bystander cells with γ-H2AX accumulated in one field of view taken at the same XY coordinates as at the time of irradiation (Figure 3 and Figure 4). Maeda et al. [28] explored the spatial distribution of dead bystander cells by using an SR X-ray microbeam. The scanned area was divided into a series of circular annuli, starting from the center. The width of each annulus was 1000 μm. In the nucleus-irradiated case that targeted the cell nucleus alone with a 10 μm × 10 μm X-ray beam, the SF of cells located at the center circle (first annulus) decreased significantly at a dose of 1.0 Gy. Bystander cell death may be induced directly by NO secreted from irradiated cells because the diffusion distance of NO in an aqueous solution is less than 1 mm. The bystander cells with pan-nuclear γ-H2AX found in the present study may be induced by NO released from the nucleus-irradiated cells with Ti_K_ X-ray microbeams. However, the molecular mechanisms underlying RIBR have not been completely elucidated.

As the International Commission on Radiological Protection (ICRP) (Ottawa, ON, Canada) described in Publication 131, the studies of cellular competition will be more important in elucidating the carcinogenic effects of low-dose and low-dose-rate radiation [22,32]. X-ray microbeams are a useful tool in radiation biology because they can precisely target cell nuclei, cytoplasm, and/or whole cells and allow the tracking and observation of cells after irradiation, although it is difficult to irradiate cells with doses close to 1 mGy, which is discussed in radiation risk assessments. Further multidisciplinary approaches are needed to clarify the biological effects of low-dose and low-dose-rate radiations.

## 5. Conclusions

X-ray microbeams are useful tools for elucidating the mechanisms underlying non-target effects, such as the RIBR that occurs under heterogeneous exposure conditions. The table-top “microbeam X-ray cell irradiation system” at CRIEPI has been successfully upgraded to enable the irradiation of Ti_K_ X-rays (4.51 keV) in addition to Al_K_ X-rays (1.49 keV). The beam size of the Ti_K_ X-rays was elliptical, with a long diameter of 7.5 μm and a short diameter of 6.9 μm. The dose rate at the sample position was approximately 0.8 Gy/min. Using this system, the nuclei of HeLa cells were successfully irradiated with high precision, and radiation-induced bystander responses were analyzed. We established a new method to quantitatively evaluate bystander cells using the fluorescence intensity of γ-H2AX as an indicator. We showed that the percentage of bystander cells with pan-nuclear induction of γ-H2AX was significantly increased in one field of view including microbeam-irradiated cells 180 and 360 min after Ti_K_ X-ray microbeam irradiation. The attenuation length (1/e) of Ti_K_ X-rays in water was approximately 171 μm, which was sufficient to irradiate cells in 3D cultured tissues. We have recently used Ti_K_ X-ray microbeams for the targeted irradiation of single intestinal stem cells in an intestinal organoid and succeeded in capturing the moment when irradiated stem cells were eliminated [22]. The upgraded microbeam cell irradiation system can be utilized to elucidate not only non-targeted effects, such as RIBR, but also cell competition in 3D cultured tissues.

## Figures and Tables

**Table 1:** Comparison of Al_K_ X-ray and Ti_K_ X-ray microbeams.

	Al_K_ X-rays	Ti_K_ X-ray
Energy (keV)	1.49	4.51
Attenuation length (1/e) in water (μm) ^1^	7.1	171
Beam size (μm) ^2^	1.8	7.4
Dose rate at the sample position (Gy/min) ^3^	84	0.8

^1^ Calculated using the X-ray database [16]. ^2^ Minimum beam size measured by knife-edge scanning. ^3^ Values under the irradiation conditions used in this study.

## Data Availability

The data presented in the current study are available from the corresponding author (M.T.) upon reasonable request.

## Supplementary Materials

The following supporting information can be downloaded at https://www.mdpi.com/article/10.3390/biology12050734/s1: Figure S1: Accumulation of 53BP1 and γ-H2AX foci in the nuclei of WI-38 cells irradiated with Al_K_ X-ray microbeams; Figure S2: Distribution of fluorescent intensity of γ-H2AX in unirradiated control cells.
