# Supplementary material for: Observation of Histone H2AX Phosphorylation by Radiation-Induced Bystander Response Using Titanium Characteristic X-ray Microbeam
Source: Biology (Basel). 2023 May 18;12(5):734. doi: 10.3390/biology12050734 (PMC10215796; doi:10.3390/biology12050734)
Supplement: Supplementary file 1 [file biology-12-00734-s001.zip › biology-2345191_Figure S1.pdf]

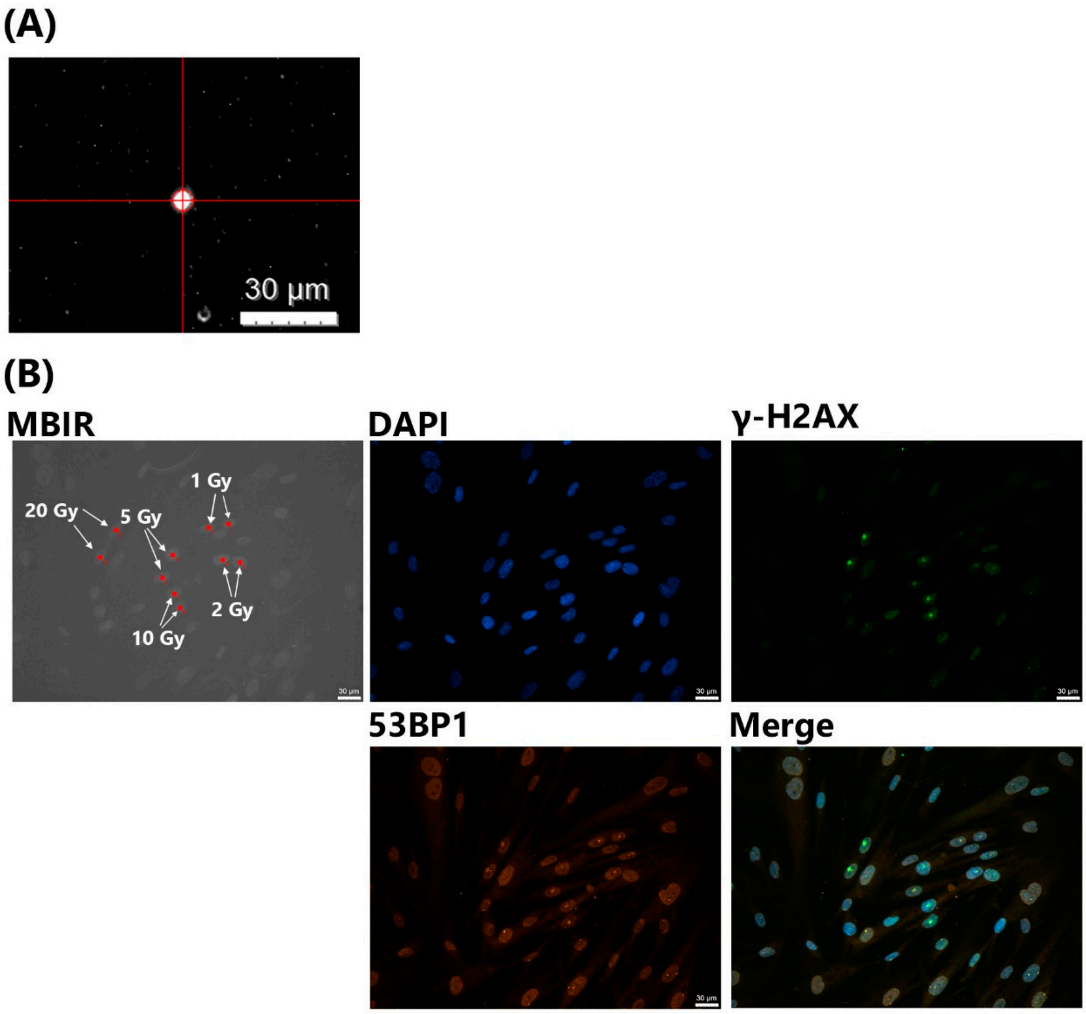

Figure S1. Accumulation of 53BP1 and γ-H2AX foci in the nuclei of WI-38 cells irradiated with Al_K X-ray microbeams. (A) An image of Al_K X-ray microbeams visualized with the scintillator. Bar represents 30 μm. (B) Co-localization of 53BP1 and γ-H2AX foci in WI-38 cells irradiated with Al_K X-ray at doses 1–20 Gy of microbeams. MBIR (microbeam irradiation) is a fluorescent image captured at the time of microbeam irradiation. Before irradiation, cell nuclei were stained with Hoechst 33258 solution for target irradiation of cell nuclei. The red squares are the irradiation positions set by the irradiation software. Cells were fixed 30 minutes after irradiation, followed by immunofluorescence. Bars represent 30 μm.
