# Supplementary material for: Observation of Histone H2AX Phosphorylation by Radiation-Induced Bystander Response Using Titanium Characteristic X-ray Microbeam
Source: Biology (Basel). 2023 May 18;12(5):734. doi: 10.3390/biology12050734 (PMC10215796; doi:10.3390/biology12050734)
Supplement: Supplementary file 1 [file biology-12-00734-s001.zip › biology-2345191_Figure S2.pdf]

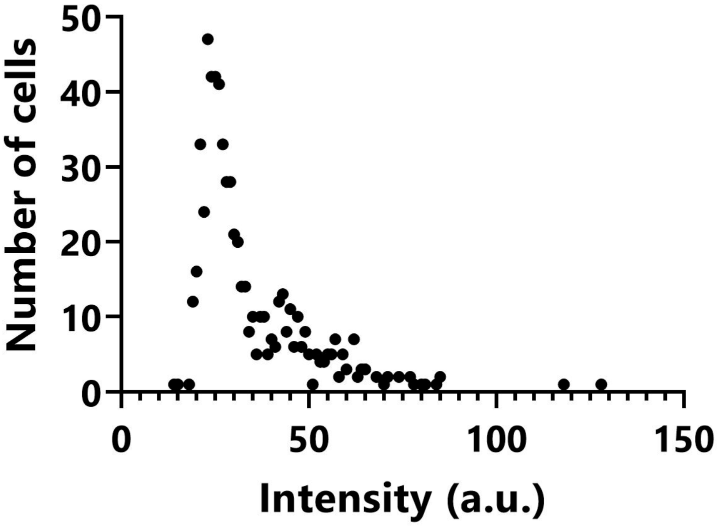

Figure S2. Distribution of fluorescent intensity of γ-H2AX in the unirradiated control cells. The fluorescent intensity was 35.01 ± 13.95. We determined that the cells with fluorescent intensity was higher than 48.96 (average + σ) were bystander cells in the irradiated dishes.
